# scKanFormer: A Transformer-KAN framework with biologically informed attention for cell type annotation in large-scale scRNA-seq data

**DOI:** 10.1371/journal.pcbi.1014607

**Published:** 2026-08-14

**Authors:** Lin Yuan, Junjie Cao, Shengguo Sun, Siguo Wang, Lan Ye, De-Shuang Huang

**Affiliations:** 1 Key Laboratory of Computing Power Network and Information Security, Ministry of Education, Shandong Computer Science Center, Qilu University of Technology (Shandong Academy of Sciences), Jinan, China; 2 Shandong Provincial Key Laboratory of Industrial Network and Information System Security, Shandong Fundamental Research Center for Computer Science, Jinan, China; 3 School of Artificial Intelligence, Zhejiang Agriculture and Forestry University, Hangzhou, China; 4 Cancer Center, The Second Qilu Hospital of Shandong University, Shandong University, Jinan, China; 5 Institute for Regenerative Medicine, Medical Innovation Center and State Key Laboratory of Cardiology, Shanghai East Hospital, School of Life Sciences and Technology, Tongji University, Shanghai, China; 6 Ningbo Key Laboratory of Multi-Omics and Multimodal Biomedical Data Mining and Computing, Eastern Institute of Technology, Ningbo, China; Duke University School of Medicine, UNITED STATES OF AMERICA

## Abstract

A key challenge in single-cell RNA sequencing (scRNA-seq) data analysis is accurately and efficiently identifying the cell type of each cell. Cell type annotation for scRNA-seq data not only needs to overcome batch effects caused by various factors but also requires effective handling of large-scale scRNA-seq datasets. Although deep learning has achieved remarkable progress in cell type annotation tasks, it still exhibits limitations in interpretability and robustness against batch effects. To tackle these issues, we propose a supervised framework based on the Transformer architecture, named scKanFormer, for cell type annotation on large-scale multi-class scRNA-seq data. To mitigate the problems of untraceable latent space, poor interpretability, and feature loss caused by the nonlinear aggregation of features in autoencoders, we employ the Transformer framework. This framework avoids dimensionality reduction and enables traceability from the attention layers back to the original input features. By integrating biological information, local and global attention mechanisms, and leveraging Kolmogorov-Arnold Networks (KAN), we enhance the model’s ability to identify and interpret cellular features. The combination of Convolutional Neural Network (CNN) and Transformer enables more comprehensive data processing, thereby mitigating batch effects. To evaluate the effectiveness and robustness of scKanFormer, we compared it with nine state-of-the-art methods on benchmark datasets. Through systematic comparisons under different cell type annotation scenarios and across various cell types, we demonstrate that scKanFormer delivers precise, robust, and transferable high-resolution annotations. These annotations are insensitive to batch effects and exhibit clear biological interpretability. The data and source code are available at https://github.com/nathanyl/scKanFormer.

## Author summary

Computational approaches for cell type annotation in scRNA-seq data have achieved notable success, yet they still face challenges in interpretability, robustness to batch effects, and effective modeling of large-scale high-dimensional data. We propose scKanFormer, a novel supervised framework for accurate and interpretable cell type annotation, built upon a Transformer architecture enhanced with Kolmogorov-Arnold Networks (KAN) and biologically informed attention mechanisms. scKanFormer constructs pathway-level representations through a knowledge-based mask matrix, enabling direct integration of prior biological information into the model. To effectively capture complex gene expression patterns, we combine global attention for modeling long-range dependencies with local attention for extracting fine-grained cellular features. Furthermore, we replace conventional fully connected layers with KAN modules, which improve interpretability through learnable spline-based activation functions, enhance continual learning capability, and provide superior representational capacity with a shallow network architecture. In addition, a convolutional neural network (CNN) module is incorporated to capture multi-scale features and strengthen robustness against batch effects. Experimental results across multiple benchmark datasets demonstrate that scKanFormer outperforms state-of-the-art methods in cell type annotation accuracy, cross-dataset generalization, and biological interpretability.

## Introduction

Single-cell RNA sequencing (scRNA-seq) has emerged as a powerful technique that allows researchers to analyze the transcriptome of individual cells with high resolution [1], and its rapid development has driven significant advances in numerous fields, such as neurology, drug development, and infectious disease research [2]. A major challenge in the analysis of scRNA-seq data is the accurate annotation of cell types for each cell. Cell type annotation addresses the cellular heterogeneity across various tissues and developmental stages, and enhances our understanding of the roles of cells and genes in tissue function and disease mechanisms [3,4].

Traditional machine learning methods have been widely applied to cell type annotation tasks [5], and these methods perform exceptionally when the data dimension is low and the sample size is small. For example, support vector machines (SVM) [6] separates different cell types by finding the optimal hyperplane. Its performance depends on parameter selection, and it incurs high computational costs for large-scale datasets. Random forest (RF) [7] is an ensemble learning method that classifies cells by constructing multiple decision trees. However, RF struggles to capture complex feature interactions. Scmap [8] annotates cell types by calculating the similarity between each target cell and each cell in the reference library. However, Scmap is sensitive to low-quality datasets and may face the curse of dimensionality when dealing with high-dimensional datasets. SingleR [9], a non-parametric method for cell annotation, relies on com-paring the gene expression patterns of target cells with bulk transcriptomes to predict cell types. SingleR is overly reliant on reference datasets, making it difficult to meet the growing demand for cell type annotation.

Recently, the continuous innovation of deep learning (DL) technology has enabled it to play an increasingly important role in cell type annotation [10]. For example, ACTINN [11] adopts the multilayer perceptron (MLP)-based model to learn higher-level representations from scRNA-seq profiles and then assigns cell type labels automatically. scBert [12], one of the most widely used cell type annotation methods, is inspired by the BERT and is pre-trained based on millions of unlabeled data from various sources to learn scRNA-seq knowledge and annotate cell types. However, this model requires large-scale pre-training data and consumes a lot of computing resources. CIForm [13] adopts a lightweight Transformer architecture and leverages self-attention to adaptively reweight feature contributions, which helps model heterogeneous cell type compositions and improves annotation accuracy. However, it lacks the integration of domain-specific expertise. TOSICA [14] modifies the traditional positional encoding in Trans-former by leveraging specific biological knowledge, further optimizing the model and improving cell annotation performance. However, it relies on the conventional global attention mechanism, which may fail to effectively capture the subtle differences between cell subtypes when processing complex datasets with high heterogeneity and local features. scGAA [15] uses a gated axial-attention Transformer to efficiently capture gene interactions for cell type annotation. scIMGCN [16] utilizes an interpretable graph convolutional network enhanced with Transformer and KAN mod-ules for accurate and explainable cell type annotation. TransAnno-Net [17] employs a Transformer-based transfer learning with self-supervised pre-training on large-scale unlabeled data for robust cell type annotation.

These methods have achieved impressive performance. Nevertheless, they still face some challenges. First, due to their complex nonlinear structures and lack of intuitive interpretability mechanisms, make it difficult to understand feature variations. Second, with the rapid increase in the number of single-cell transcriptomic datasets and the availability of multiple single-cell datasets, the challenge of integrating these datasets for cell annotation has become more common [18]. Cell annotation for single-cell transcriptomic data needs to account for batch effects arising from species, tissues, experimental protocols, and technological variations.

**Fig 1:**
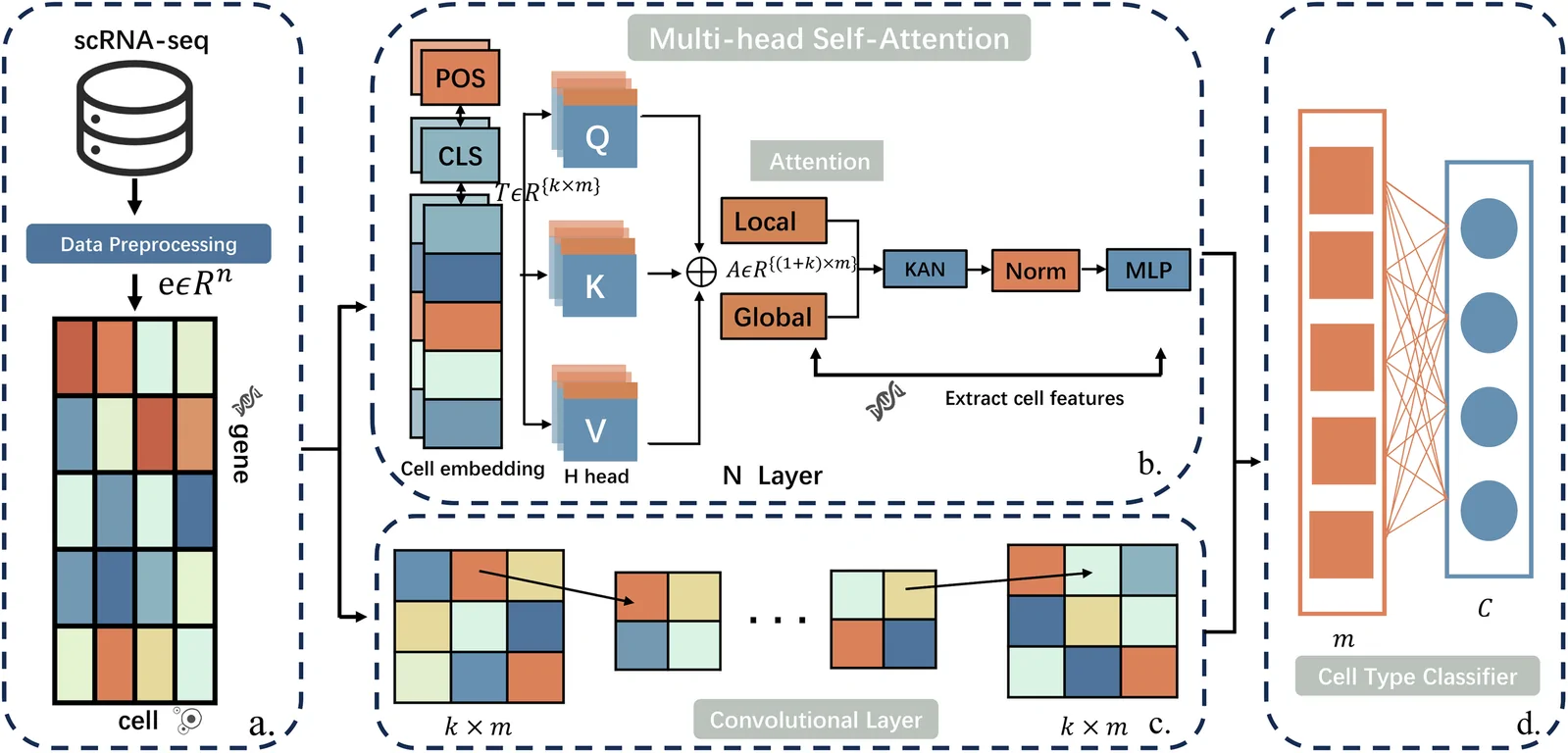
Schematic diagram of scKanFormer. a: Preprocess scRNA-seq data and input it into the model. b: After data input, expert knowledge and location encoding are combined, and the encoded data is then input into the Transformer module. In the Transformer module, the attention mechanism consists of a combination of global attention and local attention, and the final fully connected layer is replaced by a KAN module. **c:** Input into the CNN module. **d:** Output the final deep features and obtain the cell annotation results. (*n*, number of input genes; *k*, number of gene-set tokens; *m*, embedding dimension (default 48); *C*, number of cell types).

Here we propose scKanFormer, as illustrated in Fig 1, a supervised framework that combines Transformer with convolutional modules, designed to address the challenges outlined above and improve cell type annotation performance. The combination of Transformer and Convolutional Neural Network (CNN) enhances the interpretability of the model. The Transformer explicitly shows the input regions the model focuses on through the self-attention mechanism, and intuitively reveals the dependency relationships between features using attention weights, thereby making the models decision-making process more transparent and traceable. CNN [19] leverage its local perception capability to clearly exhibit the features extracted by each convolutional layer. Furthermore, scKanFormer incorporates prior biological knowledge into its attention module and operates without batch labels. By aggregating genes into pathway-level representations and combining multi-scale attention with convolutional feature extraction, scKanFormer learns batch-invariant biological patterns rather than dataset-specific noise. This design enables interpretable cross-dataset integration and cell type annotation with strong robustness to batch effects, while still preserving biological heterogeneity.

For the cell annotation task, scKanFormer introduces targeted improvements to the Transformer architecture, overcoming its limitations in the field of scRNA-seq data analysis. KAN [20] combines the feature learning ability of MLP with the local advantages of spline functions, enabling it to accurately capture complex patterns in scRNA-seq data while avoiding catastrophic forgetting [21]. Its local adjustment mechanism only modifies parameters related to new samples, preserving previously learned cellular features and ensuring that the model maintains high adaptability and stability when processing new data. This design enables KAN to exhibit higher accuracy and robustness in high-dimensional sparse scRNA-seq data analysis, making it an ideal choice for handling cellular heterogeneity and data noise. The local attention mechanism focuses on capturing dependencies between highly correlated genes or regional features, better handling sparse data while preserving key local biological characteristics [22]. In addition, global attention [23] focuses on long-range dependencies and global patterns within the entire dataset, enhancing the model’s ability to generalize to heterogeneous data. scKanFormer improves the accuracy and interpretability of cell type annotation and mitigates batch effects by combining KAN with local and global attention modules to learn discriminative representations while incorporating biological prior knowledge.

To evaluate the effectiveness and robustness of scKanFormer, we compared it with nine state-of-the-art methods on benchmark datasets (see Table 1). Through systematic comparisons under different cell type annotation scenarios and across various cell types, we demonstrate that scKanFormer provides precise, robust, and transferable high-resolution annotations that are insensitive to batch effects and offer clear biological interpretability.

**Table 1:** The detailed information of datasets.

Dataset	Protocol	Species	#Cell_num	#Cell types/subtypes	#Accession ID
**hPancreas**	inDrop/CEL-seq2/Smart-seq2	Homo Sapiens	14818	13/11	GSE84133/GSE85241/GSE81608/E-MTAB-5061/GSE86473
**hPancreas2**	CEL-seq2/inDrop	Homo Sapiens	16382	14	GSE81076/GSE85241/GSE86469/E-MTAB-5061/GSE84133/GSE81608
**hLung**	Drop-seq	Homo Sapiens	32472	17	GSE130148
**hKidney**	10xGenomics	Homo Sapiens	19985	13	GSE151302
**hRetain**	10x 3’ v2	Homo Sapiens	19694	3	E-MTAB-7316
**hOvary**	10x 3’ v2	Homo Sapiens	20676	9	GSE118127
**hBlood**	single-cell RNA sequencing/10x 5’ v2	Homo Sapiens	12175	9	PRJNA1106903
**mEmbryo**	Cell Atlas	Mus musculus	89429	4	GSE228590

## Results

### Ablation experiments

To evaluate the contributions of the core components in scKanFormer, we constructed four modified versions of the model: (i) (w/o) expert knowledge; (ii) (w/o) local attention; (iii) (w/o) CNN; (iv) KAN- > FC. (w/o) expert knowledge indicates that scKanFormer does not use expert knowledge and only uses traditional positional encoding. (w/o) local attention means that scKanFormer does not include local attention, but only uses global attention. (w/o) CNN indicates that the model excludes the CNN module. KAN- > FC means replacing the KAN module with a fully connected layer. These variant models were trained under same hyperparameter settings, allowing us to compare their performance with scKanFormer across seven benchmark single-cell datasets.

**Fig 2:**
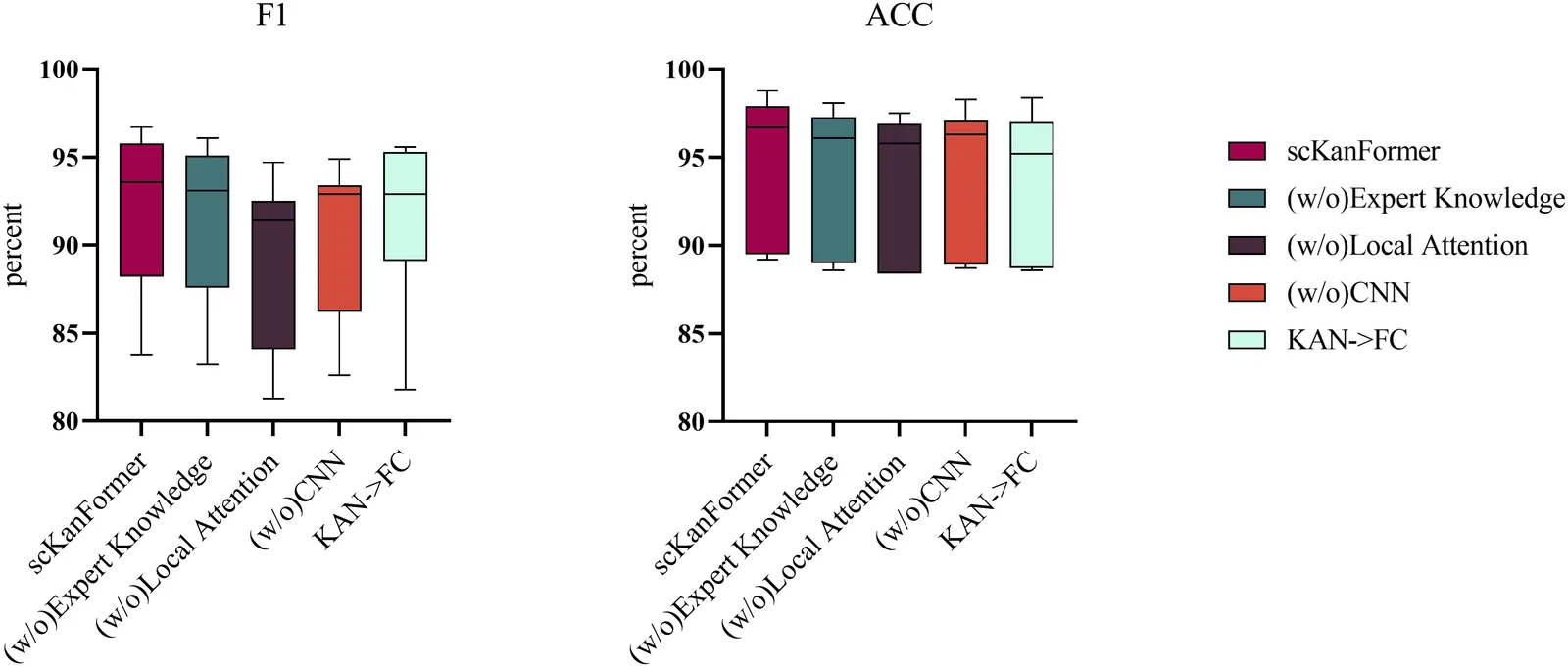
Ablation experiment results.

As illustrated in Fig 2, removing expert knowledge, local attention, CNN module, and replacing KAN with fully connected layer resulted in F1 and ACC decreases of 0.6% and 0.7%, 2.8% and 1%, 1.5% and 0.5%, and 1.90% and 0.8%, respectively. Ablation experiments demonstrate that expert knowledge, local attention, CNN module, and KAN layer all make significant contributions to the overall performance of cell type annotation.

To further investigate the effectiveness of the expert knowledge mask, we compared the expert knowledge mask with a random mask across different cell types using the F1 score. The results are presented in S1 Table. The results show that the expert masking method is superior to the random masking method, and its performance is also better than that of the random masking method in more challenging cell types.

We further evaluated the contribution of the KAN module by comparing the KAN-based model with a variant in which KAN was replaced by a standard fully connected (FC) layer. As shown in S2 Table, the KAN-based model achieved superior performance, despite requiring more parameters and longer training time. These results demonstrate that KAN contributes meaningful performance improvements, with its primary advantage stemming from enhanced representational capacity and interpretability rather than parameter or computational efficiency.

### The impact of expert knowledge on the model

**Fig 3:**
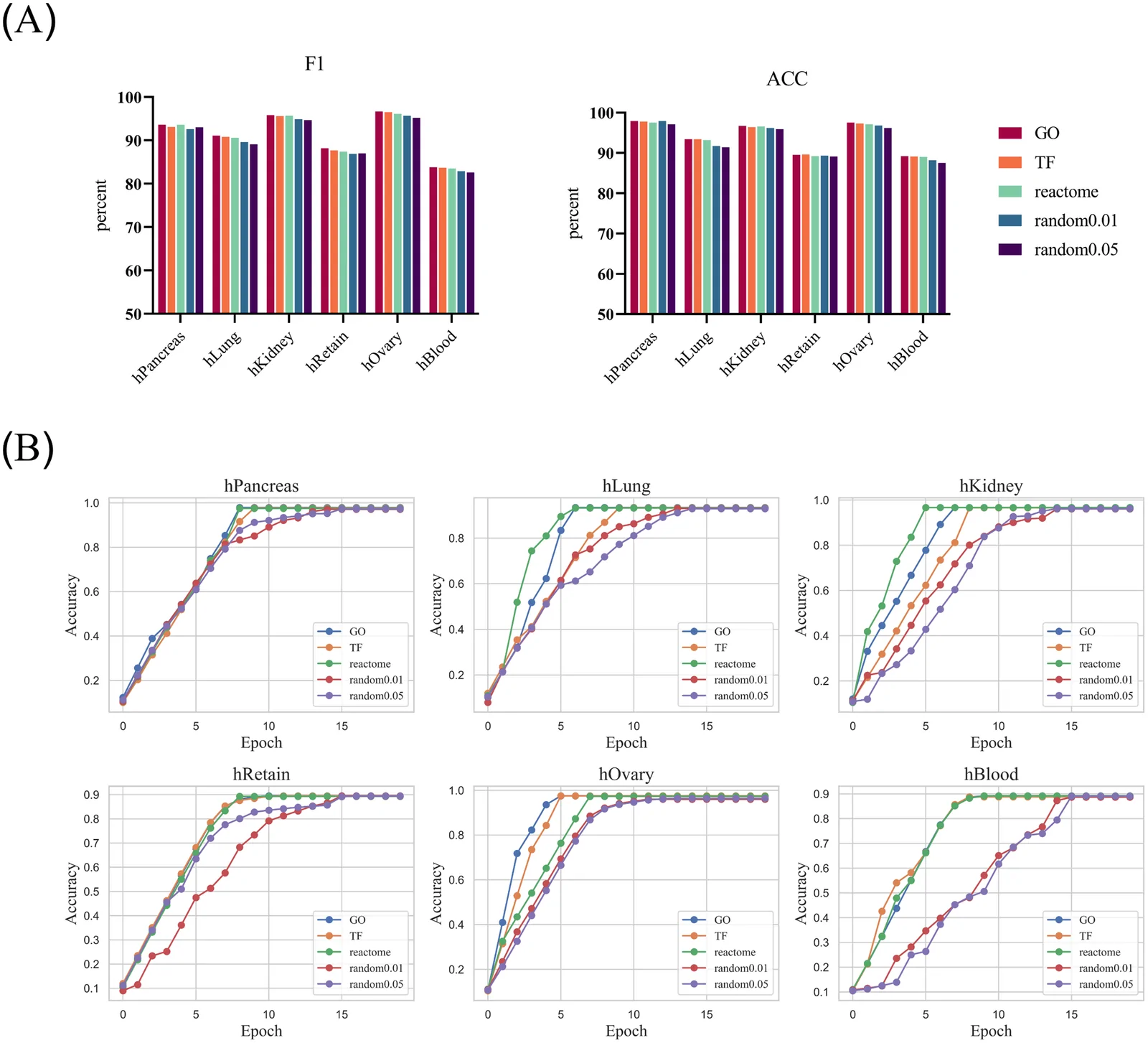
(A) The choice of mask does not affect scKanFormer’s accuracy on query dataset. **(B)** Compared to a mask based on expert knowledge, a model using a random mask with the same proportion of connections requires more epochs to converge during training.

In this section, we tested the impact of different masks on accuracy to evaluate how mask selection influences model performance. To simulate the scenario where expert knowledge is not provided, we constructed two random masks that retained 1% and 5% of the connections, respectively. This approach avoids increasing the number of parameters while maintaining the randomness of the masks as much as possible. As shown in Fig 3A, the accuracy performances of different expert knowledge masks are similar, while random masks typically lead to a decrease in model accuracy because they cannot effectively guide the model’s learning with-out domain knowledge. Furthermore, as shown in Fig 3B, models using random masks tend to require more training epochs to converge, indicating that masks lacking expert knowledge have a negative impact on the training process.

The experimental results demonstrate that the scKanFormer model is not limited to a single source of expert knowledge and exhibits strong robustness in terms of mask selection. This means that users can select an appropriate mask for cell type annotation based on specific biological contexts or research interests. Furthermore, we found that the integration of expert knowledge plays an important role in model training. Expert knowledge not only improves model accuracy but also significantly accelerates convergence speed. Specifically, when masks with expert knowledge are combined with positional encoding, the model can better capture the underlying relationships between cells, thereby improving the overall accuracy. This combination provides the model with more background information, enabling it to better understand and predict cell types.

### Performance comparison of cell type annotation methods

In this section, we compare the performance of scKanFormer with RF, SVM, SingleR, ACTINN, CIForm, TOSICA, scGAA, scIMGCN and TransAnno-Net. We merged the hPancreas dataset and the hPancreas2 dataset into a single hPancreas dataset. We evaluated all ten methods using the same collection of seven benchmark single-cell datasets, including hPancreas, hLung, hKidney, hRetain, hOvary, hBlood, and mEmbryo. We used five-fold cross-validation to calculate the F1 scores and ACC on seven datasets for these methods.

**Fig 4:**
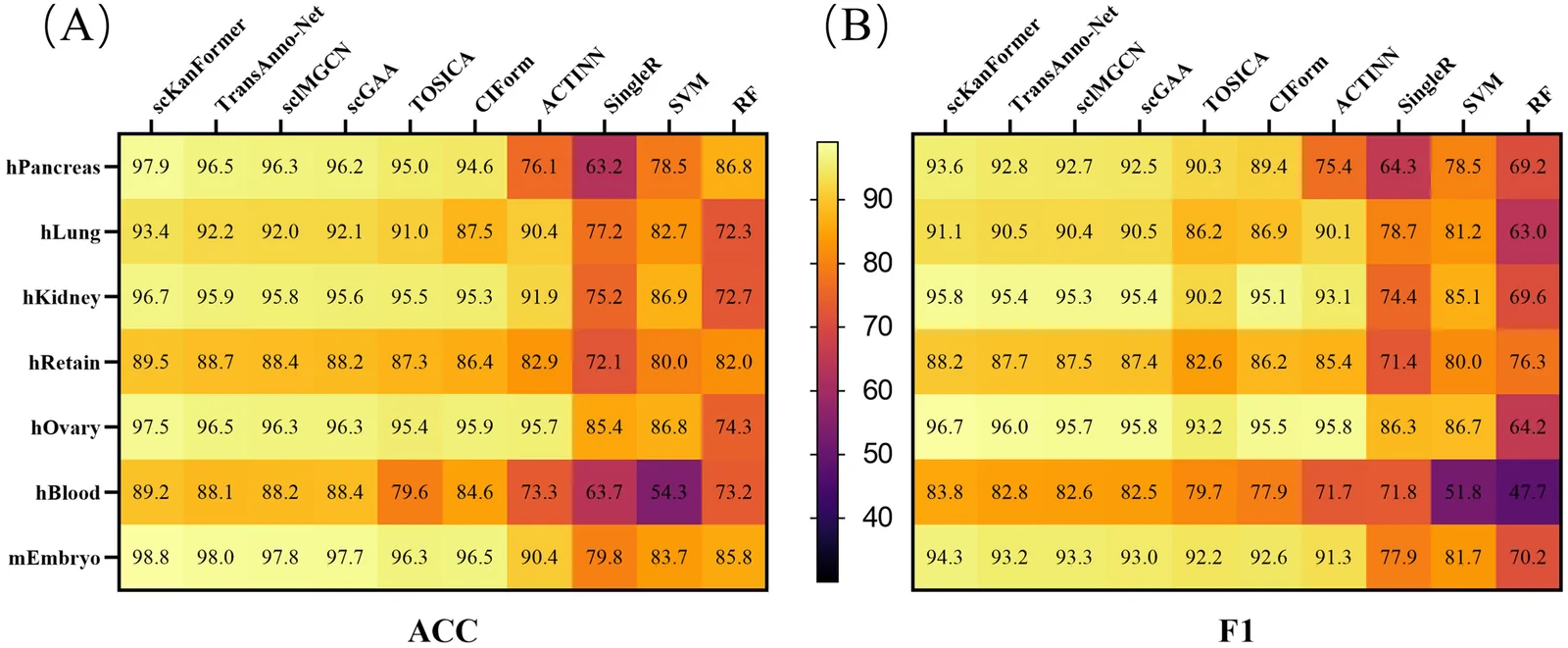
(A) Comparison of F1 between scKanFormer and nine competitive methods on seven single-cell benchmark datasets. **(B)** Comparison of ACC between scKanFormer and nine competitive methods on seven single-cell benchmark datasets.

As depicted in Fig 4, scKanFormer consistently surpasses the other methods on every dataset. Among the nine competing methods, TransAnno-Net and sclMGCN performed best. Detailed F1 and ACC were listed in Tables 2 and 3. The scKanFormer has an F1 score of 0.919, higher than TransAnno-Net’s 0.912 and scIMGCN’s 0.911. The scKanFormer has an average ACC of 0.947, surpassing TransAnno-Net’s 0.937 and scIMGCN’s 0.935.

**Table 2:** Benchmark results on seven different scRNA-seq datasets in terms of F1.

F1	hPancreas	hLung	hKidney	hRetain	hOvary	hBlood	mEmbryo
**scKanFormer**	**93.6 ± 0.7**	**91.1 ± 0.5**	**95.8 ± 0.4**	**88.2 ± 0.8**	**96.7 ± 0.3**	**83.8 ± 1.1**	**94.3 ± 0.4**
**TransAnno-Net**	92.8 ± 0.9	90.5 ± 1.4	95.4 ± 0.6	87.7 ± 1.3	96.0 ± 0.5	82.8 ± 1.8	93.2 ± 0.8
**sclMGCN**	92.7 ± 1.1	90.4 ± 1.3	95.3 ± 0.7	87.5 ± 1.4	95.7 ± 0.6	82.6 ± 1.7	93.3 ± 0.8
**scGAA**	92.5 ± 1.0	90.5 ± 1.2	95.4 ± 0.6	87.4 ± 1.4	95.8 ± 0.6	82.5 ± 1.9	93.0 ± 0.7
**TOSICA**	90.3 ± 1.2	86.2 ± 1.6	90.2 ± 1.3	82.6 ± 1.8	93.2 ± 0.8	79.7 ± 2.2	92.2 ± 0.9
**CIForm**	89.4 ± 1.3	86.9 ± 1.8	95.1 ± 0.7	86.2 ± 1.5	95.5 ± 0.6	77.9 ± 2.4	92.6 ± 1.1
**ACTINN**	75.4 ± 3.2	90.1 ± 1.1	93.1 ± 0.9	85.4 ± 1.6	95.8 ± 0.5	71.7 ± 2.5	91.3 ± 1.4
**SingleR**	64.3 ± 4.1	78.7 ± 2.8	74.4 ± 3.5	71.4 ± 2.8	86.3 ± 1.8	71.8 ± 2.7	77.9 ± 2.2
**SVM**	78.5 ± 2.5	81.2 ± 2.0	85.1 ± 1.8	80.0 ± 2.2	86.7 ± 2.1	51.8 ± 3.5	81.7 ± 1.9
**RF**	69.2 ± 3.5	63.0 ± 3.2	69.6 ± 3.8	76.3 ± 2.5	64.2 ± 3.8	47.7 ± 4.0	70.2 ± 3.0

**Table 3:** Benchmark results on seven different scRNA-seq datasets in terms of ACC.

ACC	hPancreas	hLung	hKidney	hRetain	hOvary	hBlood	mEmbryo
**scKanFormer**	**97.9 ± 0.3**	**93.4 ± 0.5**	**96.7 ± 0.4**	**89.5 ± 1.0**	**97.5 ± 0.3**	**89.2 ± 1.1**	**98.8 ± 0.2**
**TransAnno-Net**	96.5 ± 0.5	92.2 ± 0.8	95.9 ± 0.3	88.7 ± 1.3	96.5 ± 0.4	88.1 ± 1.4	98.0 ± 0.3
**sclMGCN**	96.3 ± 0.5	92.0 ± 0.9	95.8 ± 0.7	88.4 ± 1.5	96.3 ± 0.5	88.2 ± 1.7	97.8 ± 0.4
**scGAA**	96.2 ± 0.6	92.1 ± 1.0	95.6 ± 0.6	88.2 ± 1.1	96.3 ± 0.4	88.4 ± 1.5	97.7 ± 0.6
**TOSICA**	95.0 ± 0.8	91.0 ± 1.3	95.5 ± 0.9	87.3 ± 1.6	95.4 ± 0.8	79.6 ± 2.4	96.3 ± 0.9
**CIForm**	94.6 ± 1.2	87.5 ± 1.6	95.3 ± 1.1	86.4 ± 1.9	95.9 ± 0.5	84.6 ± 1.8	96.5 ± 0.7
**ACTINN**	76.1 ± 3.5	90.4 ± 1.1	91.9 ± 1.5	82.9 ± 2.3	95.7 ± 0.6	73.3 ± 2.8	90.4 ± 1.6
**SingleR**	63.2 ± 4.5	77.2 ± 2.8	75.2 ± 3.2	72.1 ± 3.4	85.4 ± 1.9	63.7 ± 3.0	79.8 ± 2.5
**SVM**	78.5 ± 2.8	82.7 ± 2.2	86.9 ± 1.9	80.0 ± 2.5	86.8 ± 2.0	54.3 ± 4.8	83.7 ± 2.1
**RF**	86.8 ± 2.2	72.3 ± 3.0	72.7 ± 3.5	82.0 ± 2.2	74.3 ± 3.5	73.2 ± 2.2	85.8 ± 2.7

### Effectiveness evaluation on intra-dataset

**Fig 5:**
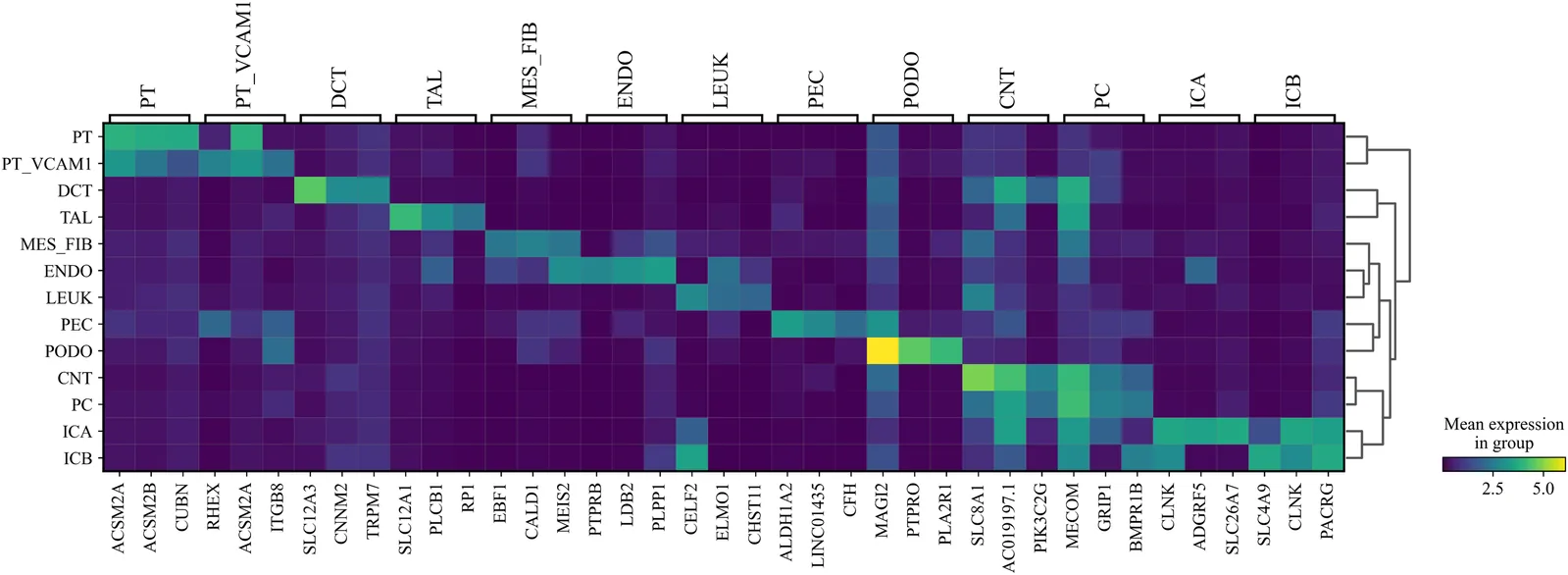
Heatmap of highly expressed genes.

In this section, we use the hKidney dataset as a case study for detailed analysis because it has a moderate number of cells and types, with a high-dimensional, multi-class, and complex feature structure. As shown in Fig 5, the dataset demonstrates clear distinctions between certain cell types, while others are more challenging to distinguish. For example, the similarity between PC and CNT cell types is relatively high.

scKanFormer effectively identifies distinct kidney cell types within the hKidney dataset, achieving superior performance in both ACC and F1-score metrics. The classification accuracy of scKanFormer for each cell type in the dataset is as follows: 98.2% for TAL, 100% for PODO, 95.6% for ICB, 94.6% for PT_VCAM1, 99.1% for PEC, 100% for LEUK, 95.6% for MES_FIB, 99.5% for ICA, 98.0% for DCT, 95.2% for ENDO, and 98.3% for PT. For the more challenging PC and CNT cell types, the ACC are 84.8% and 91.7%, respectively. scKanFormer overcomes the data imbalance existing in low-frequency and similar cell types by virtue of sophisticated feature learning and attention mechanisms, thereby successfully improving the recognition accuracy of these cell types.

**Fig 6:**
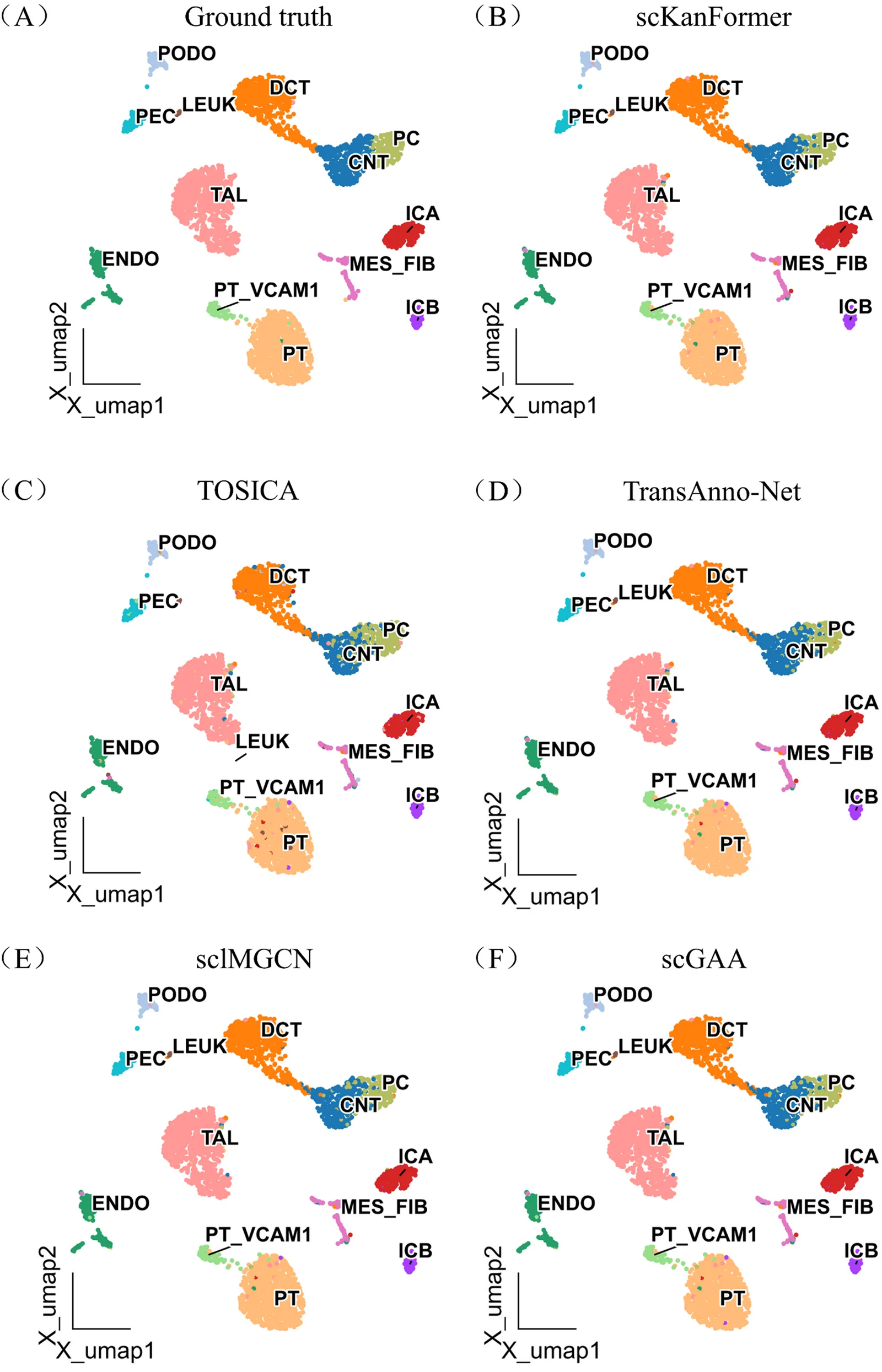
(A) The true cell type labels for the hKidney dataset. **(B)** The annotation results of scKanFormer for the hKidney dataset. **(C)** The annotation results of TOSICA for the hKidney dataset. **(D)** The annotation results of TransAnno-Net for the hKidneydataset. **(E)** The annotation results of scIMGCN for the hKidneydataset. **(F)** The annotation results of scGAA for the hKidneydataset.

The boundaries between cell types in the hKidney dataset are blurred, with many overlaps. Traditional classification methods face significant challenges, often leading to overfitting or misclassification [24]. Our proposed scKanFormer, by combining biological knowledge with an improved Transformer, can capture subtle differences between different cell types, thereby achieving fine-grained classification. With the assistance of CNNs in the model, the reliability of the classification results is further enhanced, ensuring that the prediction results for each cell type are biologically interpretable. As shown in Fig 6, compared with other competing methods, our model can more clearly delineate the boundary between PC and CNT. The annotation results are more consistent with the true cell type distribution, while the annotation results of other methods have problems such as unclear cell boundaries and fail to identify certain cell types. Our method can more effectively capture the subtle distinctions among cell types and yields more accurate annotation results.

### Effectiveness evaluation on inter-dataset

The continuous development of scRNA-seq technology has enabled different sequencing platforms to generate reference datasets and query datasets for cell type annotation. With the increasing number of datasets, the need for cross-dataset annotation is also growing. Addressing batch effects between datasets is a significant challenge in cell type annotation [25]. Furthermore, differences in the number of cell types between query datasets and reference datasets can lead to some DL-based methods (e.g., scDHA [26] and scVI [27]) failing to accurately identify cell types.

**Fig 7:**
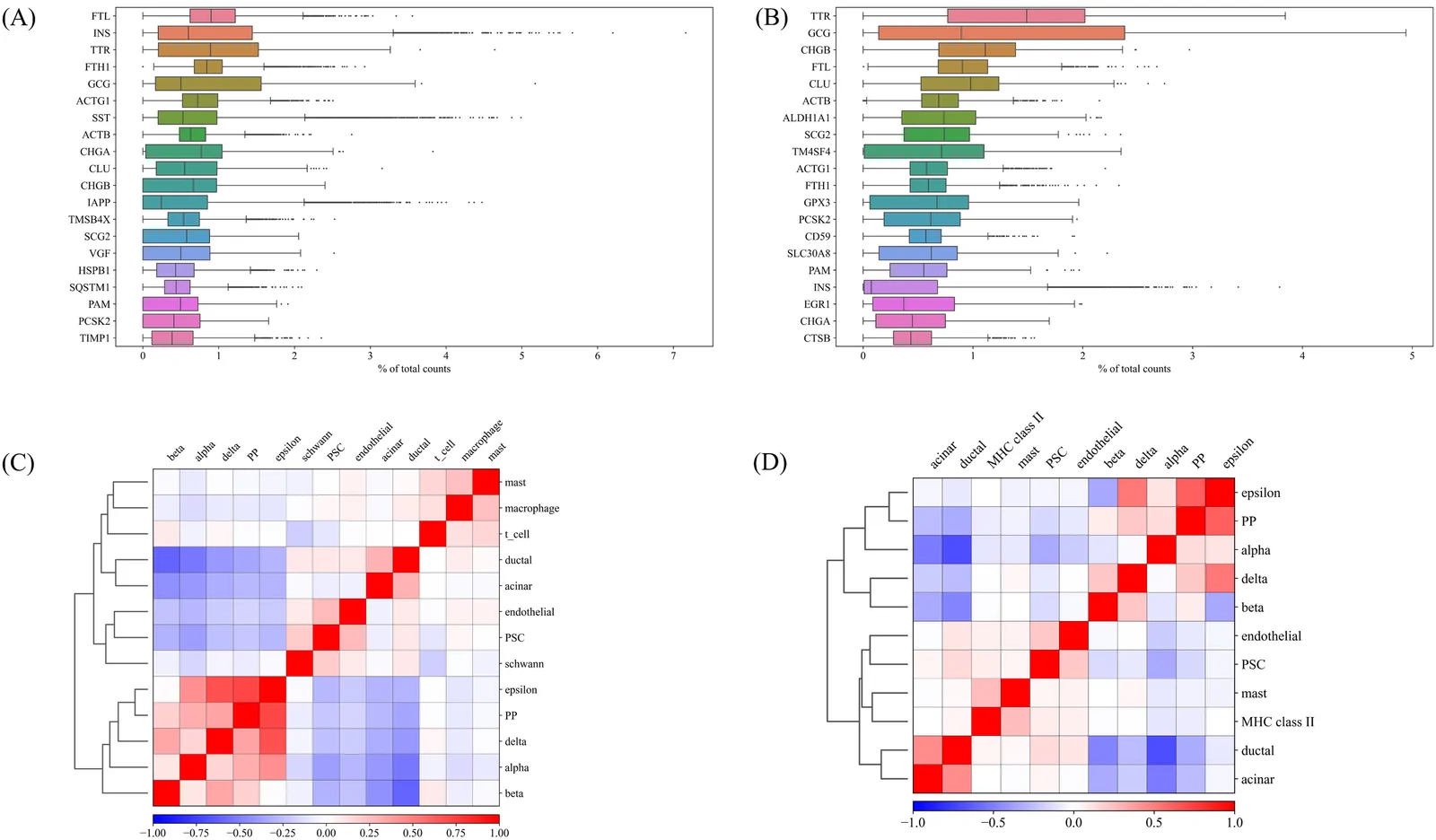
(A) Top 20 highly expressed genes in the training dataset. **(B)** Top 20 highly expressed genes in the test dataset. **(C)** The heatmap of training dataset cell type correlation. **(D)** The heatmap of test dataset cell type correlation.

We used the hPancreas dataset to evaluate the effectiveness of scKanFormer. The data in this dataset is generated by three sequencing methods (inDrop [28], CEL-seq2 [29], and Smart-seq2 [30]), and there are differences in cell types between the reference and query datasets. we analyzed the correlation between highly expressed genes and cell types in the reference and query datasets. As shown in Fig 7, we observe that there are significant differences in the distribution of highly expressed genes between the two datasets, indicating that there are differences in the number of cell types between the two datasets.

**Fig 8:**
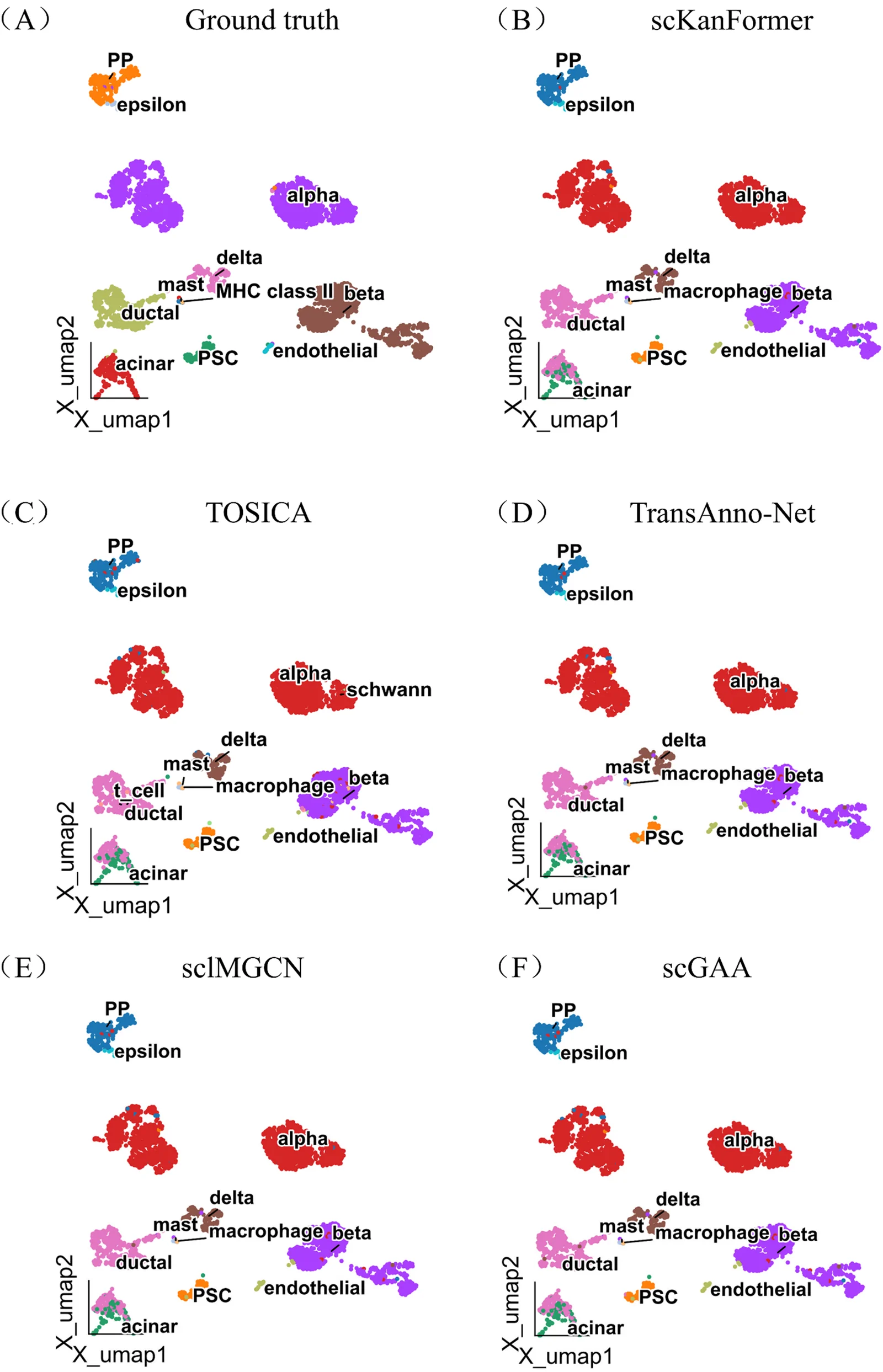
(A) The true cell type labels for the hPancreas dataset. **(B)** The annotation results of scKanFormer for the hPancreas dataset. **(C)** The annotation results of TOSICA for the hPancreas dataset. **(D)** The annotation results of TransAnno-Net for the hPancreas dataset. **(E)** The annotation results of scIMGCN for the hPancreas dataset. **(F)** The annotation results of scGAA for the hPancreas dataset.

We used this dataset to evaluate the performance of scKanFormer in annotating cell types. We further used UMAP [31,32] dimensionality reduction plots to illustrate the classification results of scKanFormer and other models. As shown in Fig 8, the dimensionality reduction clustering plot of TOSICA shows 13 cell types, indicating that its generalization ability is insufficient and the model is not robust to handling distributional differences between datasets. In the plots of TransAnno-Net and scIMGCN, endothelial includes other cell types. In the scGAA annotation results, alpha includes other cell types.

Compared to competing methods, the number and distribution of cell types annotated by scKanFormer closely resemble the ground true labels, demonstrating strong generalization ability and accurate data distribution capture. Although scKanFormer can handle complex single-cell data and provide high-quality annotation results, there are still a few instances of misclassified cell types. These misclassifications may stem from the fact that these types of cells are too few in number and highly similar to other cell types, leading to insufficient feature learning by the model.

### Evaluation of Multi-Dataset Integration Effectiveness

**Fig 9:**
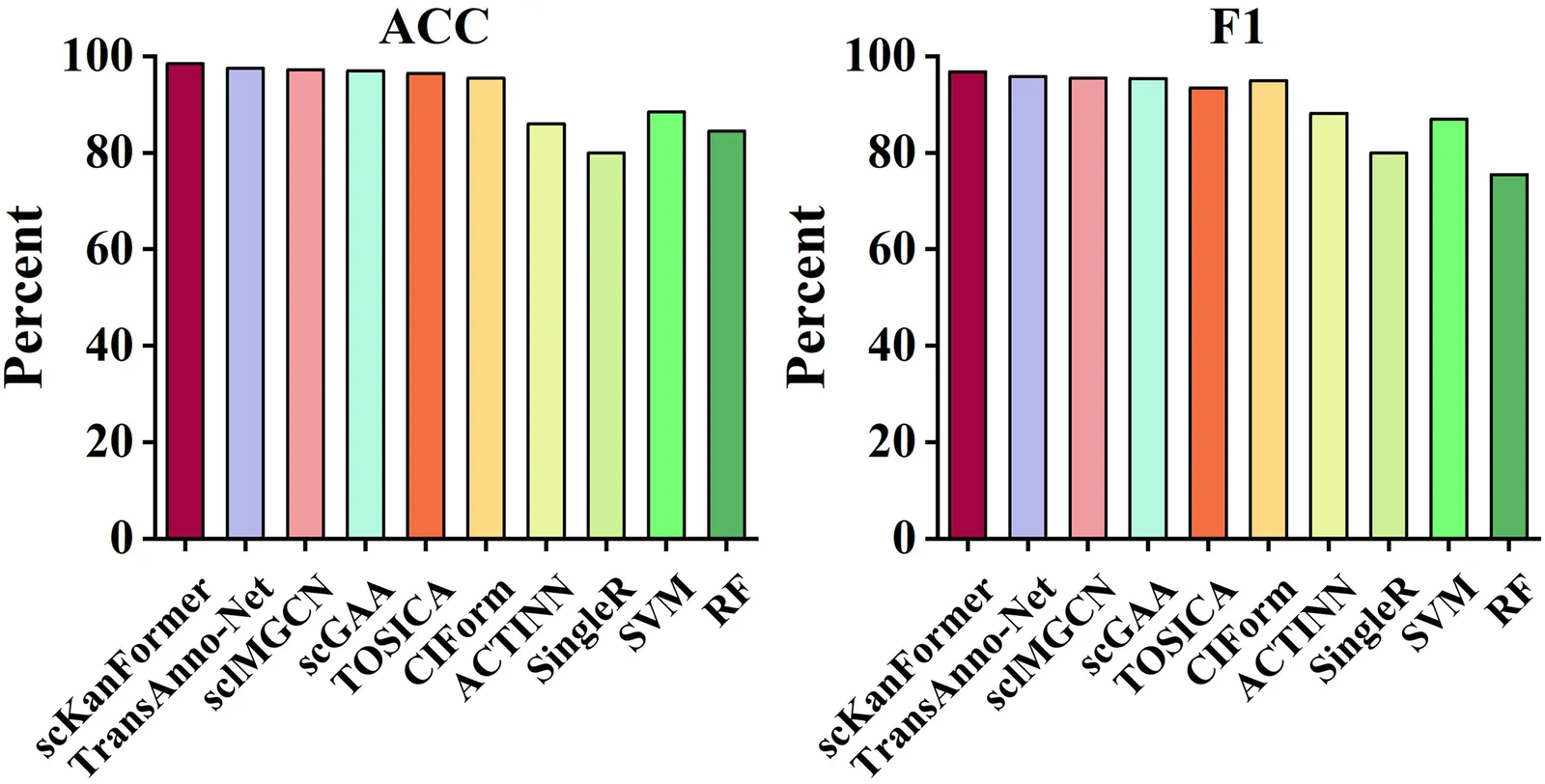
hPancreas2 experiment results.

With the continuous development of single-cell sequencing technology and the increasing number of datasets, new demands have been placed on the ability of models to achieve precise annotation after integrating multiple datasets. We tested the model’s performance on hPancreas2 and hBlood. The hPancreas2 dataset consists of six sub-datasets, and hPancre-as2 contains four identical datasets as hPancreas. We mixed these six sub-datasets and then divided them into reference and query datasets. As shown in Fig 9, our model outperforms other competitive methods in both ACC and F1 score, demonstrating its ability to effectively overcome batch effects and maintain stable performance when integrating multiple datasets.

**Fig 10:**
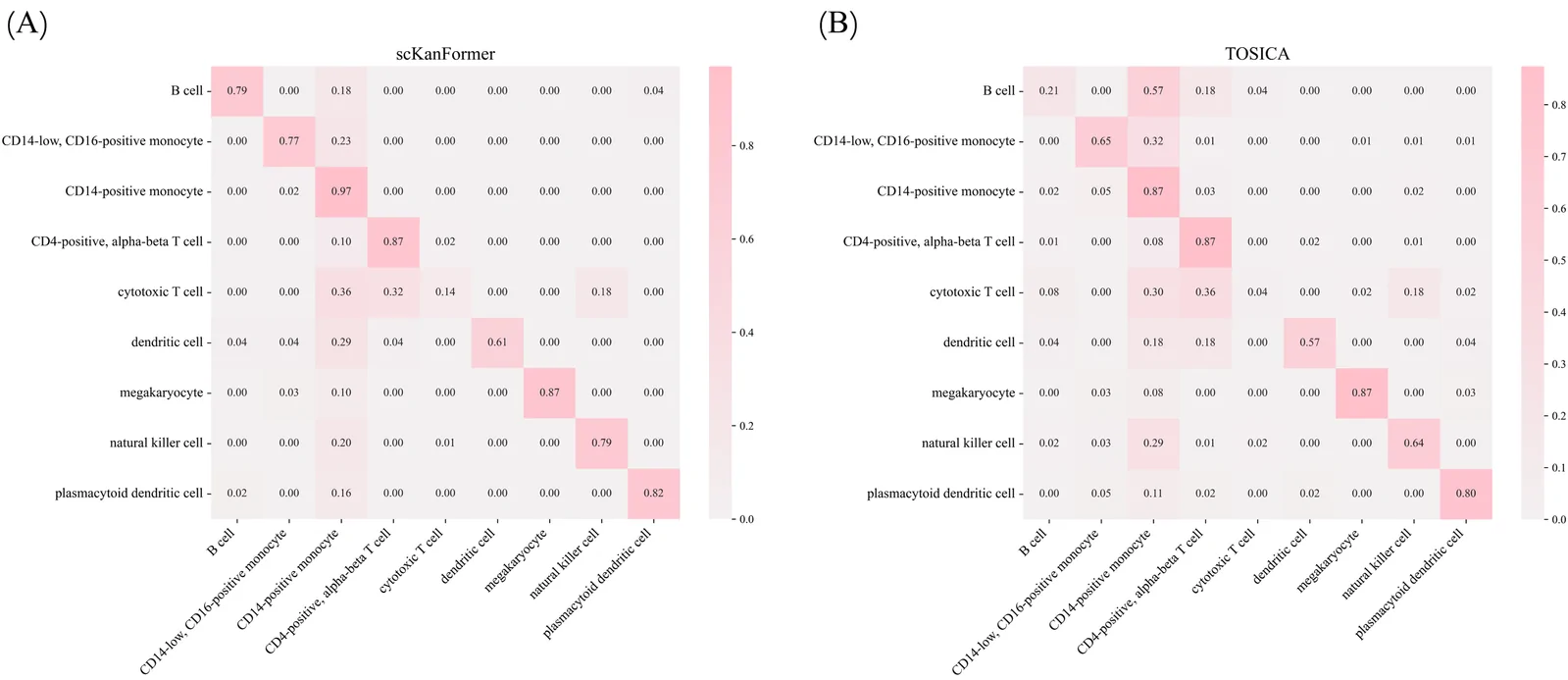
(A) Confusion matrix of scKanFormer prediction on hBlood. **(B)** Confusion matrix of TOSICA prediction on hBlood.

As shown in Fig 10, on the hBlood dataset, we compared the cell type annotation results of scKanFormer and TOSICA. scKanFormer achieved higher annotation accuracy for most cell types than TOSICA. This indicates that the model has a stronger capability in cell type identification.

## Discussion

In this paper, we propose a supervised framework named scKanFormer, which combines Transformer with KAN module to improve the performance of cell type annotation. The integration of Transformer and CNN enhances the interpretability of the model. KAN combines the feature learning capability of MLP with the local advantages of spline functions, enabling it to accurately capture complex patterns in scRNA-seq data while avoiding catastrophic forgetting. The use of the attention mechanism strengthens the model’s generalization ability on heterogeneous data. To evaluate the effectiveness and robustness of scKanFormer, we compared it with nine state-of-the-art methods on benchmark datasets. Through systematic comparisons under different cell type annotation scenarios and across various cell types, we demonstrate that scKanFormer provides precise, robust, and transferable high-resolution annotations that are insensitive to batch effects and offer clear biological interpretability.

Compared with existing methods, the high accuracy and robustness of scKanFormer stem from the attention layers and markers in the Trans-former architecture that are masked by biologically relevant pathways or regulators. This design directs the model’s attention toward biologically meaningful interactions among genes, pathways, and regulatory elements, instead of relying on isolated genes that are easily disrupted by technical noise or batch variations. As a result, scKanFormer can accurately identify known cell types and high-resolution subtypes with improved resolution, while extracting biologically meaningful dynamic behavioral patterns from the data. The local attention mechanism fine-tunes the capture of interactions between cells by focusing on local relationships, whereas the global attention mechanism ensures that the model can fully understand the global structure in large-scale datasets, enhancing its ability to perform cell type annotation. The integration of KAN and CNN not only improves scKanFormer’s ability to handle complex data but also significantly boosts the model’s sensitivity to biological structures, enhancing its ability to capture multi-scale features and improving its generalization across diverse datasets and experimental conditions.

Despite these advances, scKanFormer has several limitations that warrant further investigation. The self-attention mechanism scales quadratically with the number of pathway tokens, which may increase memory consumption for large gene-set collections; future work could explore sparse or linear attention to improve scalability. The knowledge-based mask relies on the completeness of prior pathway annotations, which may be limited for non-model organisms or poorly characterized tissues. Furthermore, rare cell types and highly similar subtypes remain challenging to annotate accurately due to limited training samples and subtle transcriptional differences, suggesting a need for data augmentation or contrastive learning strategies. When query data contain genuinely unseen cell types, the model may misclassify them into the most similar known category.

In the future, effectively identifying novel cell types through open-set recognition or other adaptive strategies remains an important direction for our future work. In addition, we plan to integrate multimodal data – including spatial transcriptomics, proteomics, and epigenomics - to further improve annotation performance for challenging cell populations.

## Materials and methods

### Data preparation

To assess scKanFormer, we collected and curated eight benchmark scRNA-seq datasets covering two species (human and mouse) and seven tissues: Pancreas, Lung [33], Kidney [34], Retina [35], Ovary [36], Blood [37], Embryo [38]. In addition, there is a special dataset Pancreas2. The information for these datasets is listed in Table 1.

The pancreas dataset focuses on the human pancreas (hPancreas) and consists of five datasets: Baron (GSE84133), Muraro (GSE85241), Xin (GSE81608), Segerstolpe (E-MTAB-5061), and Lawlor (GSE86473). The first three datasets constitute the training dataset, and the latter two datasets constitute the testing dataset. The training dataset contains 13 cell types, and the testing dataset contains 11 cell types. hPancreas2 is com-posed of six datasets: GSE81076, GSE85241, GSE86469, E-MTAB-5061, GSE84133, and GSE81608. We divided the combined dataset into training and testing datasets, which includes a total of 16,382 cells across 14 cell types. The human lung dataset (hLung) is from GSE130148, containing 32,472 cells and 17 cell types. The human kidney dataset (hKid-ney) is from GSE151302, with 19,985 cells and 13 cell types. The human retina dataset (hRetina) comes from E-MTAB-7316, including 19,694 cells and 3 cell types. The human ovary dataset (hOvary) is from GSE130148, encompassing 20,676 cells and 9 cell types. The human blood dataset (hBlood) is derived from two sequencing methods of the PRJNA1106903 project: single-cell RNA sequencing and 10x 5’v2 sequencing, containing 12,175 cells and 9 cell types. The mouse embryo dataset (mEmbryo) is from GSE228590, with 89,429 cells and 4 cell types.

For within-dataset experiments, original cell type annotations were used directly. For cross-dataset experiments (e.g., hPancreas), cell type labels from different sources were mapped to a common ontology following the protocol established in prior benchmark studies, ensuring that labels with equivalent biological meanings were unified before model training and evaluation.

### Data preprocessing

The scRNA-seq data is provided as a preprocessed AnnData object. Prior to model input, the expression matrix is converted from sparse to dense format, and cell type labels are encoded into numerical indices using label encoding. To mitigate class imbalance, we apply a balanced sampling strategy that downsamples each cell type to a maximum threshold before splitting the data into training (70%) and validation (30%) sets. Standard scRNA-seq preprocessing steps, including cell filtering, gene filtering, normalization, log-transformation, and scaling, are performed by the user prior to model input using standard pipelines such as Scanpy [39], ensuring flexibility across datasets with different characteristics.

### Knowledge-based mask matrix

Prior to the data being utilized by the scKanformer, a knowledge-based mask matrix must be constructed. In this study, the mask matrix is derived from the GSEA knowledge dataset (http://www.gsea-msigdb.org/gsea/downloads.jsp). The input genes are assigned to curated gene sets defined in GMT format (e.g., c2.cp.reactome.v7.5.1.symbols.gmt and c3.all.v7.5.1.symbols.gmt). Two configurable settings are available: (i) the upper bound on the number of genes retained for each gene set (default: 300) and (ii) the maximum number of gene sets (default: 300). The mask matrix is represented as a binary matrix M, where columns denote gene sets and rows represent genes. If gene *i* belongs to gene set *j*, $M_{i,j}$ is set to 1; otherwise, it is set to 0. The matrix is then stacked m (embedding dimension) times to create gene set tokens from gene inputs, with *m* is customizable with a default value of 48.

### Structure of scKanFormer

scKanFormer is a deep learning framework consisting of three primary modules: a cell embedding layer, a KAN+Transformer encoder, and a classification layer [40]. The cell embedding layer stores the position or temporal information of sub-vectors. This is crucial because the useful information carried by the sub-vectors will ultimately influence the results. As the core component of the model, the KAN+Transformer encoder utilizes self-attention layer combined with KAN to process sub-vectors, extract deep-level features from cell genes, and then integrates with CNN. The classification layer receives the output of the Transformer encoder and provides a predicted cell type. The model architecture is illustrated in Fig 1.

#### Cell mbedding layer.

In the first module (cell embedding layer) of scKanFormer, genes are transformed into tokens. The projection layer starts from a fully connected weight matrix and is subsequently constrained by a knowledge-derived mask (e.g., gene-pathway associations). After masking, only the permitted sparse gene-pathway links are kept for training, so that each resulting token aggregates signals from a specific subset of genes and serves as a pathway-level representation. This tokenization is performed in parallel for *m* times and the *m* token vectors are concatenated. Finally, a learnable class token (CLS) is added to the token sequence. This class token will extract information in subsequent neural network layers for cell type prediction. Furthermore, to preserve the positional information of input tokens, scKanFormer retains the traditional Transformer positional encoding to help the model understand the position of each token in the input sequence. This architectural design combines the traditional positional encoding with an expert knowledge-driven sparse connection mechanism, which not only enhances the model’s learning efficiency but also improves its performance and interpretability in cell type annotation tasks [41].

For each cell, the expression levels of n cellular genes ($e\in R^{n}$) are first embedded into k markers ($t\in R^{k}$) through a linear transformation with weight matrix W, where the weight matrix W is learned during the training process. A mask matrix M is generated based on expert knowledge, consisting of 0s and 1s, and having the same dimensions as weight matrix W. The masked linear transformation weight matrix $W^{\prime }$ is obtained by element-wise multiplying W and M.


$$\begin{array}{c} t=W^{\prime }\cdot e \end{array}$$ (1)

The embedding operation is then repeated in parallel *m* times to increase the dimension of the embedding space, where *m* is a hyperparameter with a default value of 48. The resulting $t_{s}$ are then concatenated column-wise.


$$\begin{array}{c} T=\text{ columnbind }\left(t_{\text{1}},t_{\text{2}},\ldots ,t_{m}\right),T\in R^{k\times m} \end{array}$$ (2)

where T represents the pathway token matrix. Each row (token) in the matrix represents a pathway.

Sine functions are applied to positions with odd indices across the tokens, while cosine functions handle those with even indices.


$$\begin{array}{c} PE(l,\text{2}i)=sin\left(l/\text{1}\text{0}\text{0}\text{0}{\text{0}}^{\text{2}i/s}\right) \end{array}$$ (3)

$$\begin{array}{c} PE(l,\text{2}i+\text{1})=cos\left(l/\text{1}\text{0}\text{0}\text{0}{\text{0}}^{\text{2}i/s}\right) \end{array}$$ (4)

where l denotes the position of $k_{ij}[l]$ within $k_{ij}$, 2i represents the even-indexed sub-vectors, and 2i+1 represents the odd-indexed sub-vectors.

These functions add essential position information to gene expression vectors, enhancing the model’s ability to predict cell types.


$$\begin{array}{c} T_{pos}=T+PE \end{array}$$ (5)

Although gene-set tokens do not have a natural sequential order like words in a sentence, positional encoding is introduced to provide distinguishable token identities for the attention mechanism. Next, a learnable parameter class token (CLS) is concatenated row-wise on top of $T_{pos}$ to obtain the input matrix I.


$$\begin{array}{c} I=rowbind(CLS,T_{POS}),CLS\in R^{m},I\in R^{\text{1}+k\times m} \end{array}$$ (6)

Ultimately, a gene-pathway mask matrix was constructed based on expert knowledge. By combining the gene-pathway mask matrix with the gene-position mask matrix, richer potential key information can be provided for subsequent model, enabling more accurate cell type annotation.

#### Transformer layer and KAN layer.

In this work, we combine the self-attention with the KAN layer to process the token representations. The attention mechanism emphasizes the most relevant tokens, helping the model prioritize essential information. While capturing global information, some important genes in scRNA-seq data play a significant role in cell type annotation tasks. Therefore, we further enhance the model’s ability to learn local information by improving the self-attention network. We incorporate a local attention mechanism to capture feature information and short-range dependencies within local regions, which helps identify subtle feature variations within cells. The local window size is set to 16 by default, partitioning the k pathway tokens into segments within which attention is computed independently. Local attention is particularly effective for distinguishing highly similar cell subtypes, which may differ only in a limited set of marker genes or pathway activities. Global attention captures relationships across the full token sequence but may dilute these subtle local signals. By computing attention independently within each local window, the model amplifies fine-grained transcriptional differences that are critical for resolving closely related cell types. We combine the global information with local information to generate the final representation. In the multi-head self-attention layer, the query (Q), key (K), and value (V) matrix are independently obtained via separate linear transformations of the input matrix I, where the corresponding projection weights are denoted as $W_{q,k,v}$.


$$\begin{array}{c} Q,K,V=W_{q,k,v}I \end{array}$$ (7)

$$\begin{array}{c} A_{\text{global }}=softmax\left(\frac{QK^{T}}{\sqrt{d_{k}}}\right)\cdot V \end{array}$$ (8)

where $Q,K,V\in R^{\text{1}+k\times m}$, $d_{K}=m$, and $A_{\text{global }}$ is the global attention mechanism.

For a given window of size local_window_size, the local attention for each window is computed as:


$$\begin{array}{c} A_{\text{local },i}=softmax\left(\frac{Q_{\text{win}}K_{\text{win}}^{T}}{\sqrt{d_{k}}}\right)\cdot V_{\text{win}} \end{array}$$ (9)

The final output A is a weighted combination of the global and local attention:


$$\begin{array}{c} A=A_{global}+A_{local} \end{array}$$ (10)

To address the issues of excessive parameters and poor interpretability of Transformer when processing high-dimensional gene expression data, we replaced the traditional feed-forward network (FFN/MLP) after the multi-head self-attention operation in each Transformer encoder block with Kolmogorov-Arnold Networks (KAN).


$$\begin{array}{c} f(x)=\sum_{q=\text{1}}^{\text{2}n+\text{1}}\Phi_{q}\left(\sum_{p=\text{1}}^{n}\varphi_{q,p}\left(x_{p}\right)\right) \end{array}$$ (11)

where f(x) is a KAN function, $\varphi_{q,p}\left(x_{p}\right)$ are spline functions, and $\Phi_{q}$ are transformations.


$$\begin{array}{c} \varphi (x)=w(b(x)+spline(x)) \end{array}$$ (12)

where φ(x) denotes activation function and w is the weight, b(x) is the basis function, implemented as silu(x) (Sigmoid Linear Unit).


$$\begin{array}{c} b(x)=silu(x)=\frac{x}{\left(\text{1}+e^{-x}\right)} \end{array}$$ (13)

KAN avoids using linear weight matrices through learnable edge activation functions and spline functions, adopting a compact architecture where the narrower hidden dimension is compensated by learnable spline basis functions for superior representational capacity. At the same time, the internal structure of KAN is easier to visualize and interpret, enhancing the model’s interpretability. Furthermore, KAN possesses continual learning capabilities, enabling the model to maintain stability and performance when handling dynamically changing cell data. Therefore, replacing fully connected layers with KAN not only improves the accuracy of cell type annotation but also offers significant advantages in terms of complexity, interpretability, and continual learning ability.

We input the original cell features without the Transformer transformation into the CNN layer.


$$\begin{array}{c} \;f(x)\;=Drop(Conv\text{2}(Drop(Conv\text{1}(x)))) \end{array}$$ (14)

scRNA-seq data contains rich multi-scale information. By combining Transformer and CNN, scKanFormer can effectively capture multi-scale features. The global attention mechanism ensures that the model can ac-count for correlations between all genes, while local attention mechanism enhances the model’s sensitivity to detailed features. Furthermore, leveraging CNNs, which excel at handling high-dimensional data, convolution operations can extract fine-grained features and genes within local regions. These features are crucial for identifying gene expression patterns in specific cell types. By combining attention mechanisms with various receptive fields and CNNs, we achieve multi-dimensional extraction and integration of gene information, thereby effectively enhances the model’s ability to capture features, better capturing complex gene expression patterns, and thus improve the accuracy and reliability of cell type annotation results [42].

#### Cell classification layer.

The classifier takes the representation generated by the classification module as input, passes it to the fully connected layer, and applies a soft-max function to generate the cell type prediction probability [43]. To improve the model’s ability to capture complex patterns, we added two fully connected layers after the attention module.

### Model training

We divided the data into training and testing sets, and reserved 30% of the training data as a validation set. The training loss was computed using cross-entropy [44]. Optimization relied on the AdamW optimizer, paired with cosine annealing algorithm [45]. All experiments were conducted on an NVIDIA RTX 4070 Ti GPU. To mitigate overfitting, we employed R-drop regularization with a dropout of 0.3 [46], a batch size of 64, and an initial learning rate of 0.001. And the embedding dimension was set to 48, as higher values (e.g., 128 or 256) increased memory consumption without proportional accuracy gains. The maximum number of gene sets was capped at 300 to control the computational cost of self-attention while retaining the most informative pathways.

### Model prediction

During inference, the trained scKanFormer model receives query cell expression profiles as input. The expression matrix is first transformed into pathway-level token representations through the knowledge-based mask matrix, then processed by the Transformer-KAN-CNN architecture. Finally, the CLS token representation is extracted and passed through the classification layer to produce cell-type probability predictions.

### Evaluation strategies

We assessed model performance using k-fold cross-validation. The model was evaluated with two metrics: accuracy (ACC), and F1-score.


$$\begin{array}{c} \left\{ \begin{array}{c} \text{ Precision }=\frac{\text{TP}}{\text{TP}+\text{FP}}\\ \text{ Recall }=\frac{\text{TP}}{\text{TP}+\text{FN}}\\ \text{ACC}=\frac{\text{TP}+\text{TN}}{\text{TP}+\text{TN}+\text{FP}+\text{FN}}\\ \;F\text{1}=\frac{\text{2}\times \text{ Precision }\times \text{ Recall }}{\text{ Precision }+\text{ Recall }} \end{array}\right. \end{array}$$ (15)

We compared the performance of scKanFormer with scGAA, scIMGCN, TransAnno-Net, TOSICA, CIForm, ACTINN, SingleR, RF, and SVM. These methods include three shallow learning methods (SingleR, RF, SVM) and six state-of-the-art DL-based methods (scGAA, scIMGCN, TransAnno-Net, TOSICA, CIForm, ACTINN). SingleR identifies cell types by comparing the gene expression pattern of a single cell with each classifier. ACTINN employs the MLP to capture higher-order patterns and perform cell type assignment. CIForm incorporates Transformers for cell annotation. TOSICA integrates Transformers with biological information for the cell annotation task. scGAA uses a gated axial-attention Transformer to efficiently capture gene interactions for cell type annotation. scIMGCN utilizes an interpretable graph convolutional network enhanced with Transformer and KAN modules for accurate and explainable cell type annotation. TransAnno-Net employs a Transformer-based transfer learning with self-supervised pretraining on large-scale unlabeled data for robust cell type annotation. We executed the competing methods using their default parameters.

### Implementation details

A fixed random seed (GLOBAL_SEED = 1) was used across all experiments to ensure reproducibility. All experiments were conducted on an NVIDIA RTX 4090D GPU. The data and source code are available at https://github.com/nathanyl/scKanFormer.

## Supporting information

S1 Table Comparison of the expert knowledge mask and a random mask across different cell types using the F1 score on the hKidney dataset. (DOCX)

S2 Table Comparison of KAN and FC variants of scKanFormer on the hPancreas dataset. (DOCX)
