# Supplementary material for: scKanFormer: A Transformer-KAN framework with biologically informed attention for cell type annotation in large-scale scRNA-seq data
Source: PLoS Comput Biol. 2026 Aug 14;22(8):e1014607. doi: 10.1371/journal.pcbi.1014607 (PMC13489525; doi:10.1371/journal.pcbi.1014607)
Supplement: Supplementary file 1 [file pcbi.1014607.s001.docx]

**Supplementary Table 1.** Comparison of the expert knowledge mask and a random mask across different cell types using the F1 score on the hKidney dataset.

| **Cell Type** | **Expert Mask F1 (%)** | **Random Mask F1 (%)** |
| --- | --- | --- |
| PODO | 100.0 | 98.5 |
| LEUK | 100.0 | 99.2 |
| PEC | 99.0 | 97.2 |
| TAL | 98.1 | 97.8 |
| PT | 98.4 | 98.5 |
| DCT | 97.9 | 97.3 |
| ICA | 99.5 | 98.1 |
| ENDO | 94.9 | 93.4 |
| MES_FIB | 95.6 | 93.8 |
| ICB | 95.1 | 91.3 |
| PT_VCAM1 | 94.4 | 91.8 |
| CNT | 90.4 | 86.5 |
| PC | 83.1 | 78.2 |
