# Supplementary material for: scKanFormer: A Transformer-KAN framework with biologically informed attention for cell type annotation in large-scale scRNA-seq data
Source: PLoS Comput Biol. 2026 Aug 14;22(8):e1014607. doi: 10.1371/journal.pcbi.1014607 (PMC13489525; doi:10.1371/journal.pcbi.1014607)
Supplement: Supplementary file 2 [file pcbi.1014607.s002.docx]

**Supplementary Table 2.** Comparison of KAN and FC variants of scKanFormer on the hPancreas dataset.

|  | **KAN** | **FC** |
| --- | --- | --- |
| Parameter quantity (single layer) | 23,040 | 18,432 |
| Training time (10 epochs) | 77min55s | 56min32s |
| F1 | 93.6 | 91.9 |
| ACC | 97.9 | 94.9 |
